# Pyoderma gangrenosum complicated with hematological malignancies: Two case reports

**DOI:** 10.1097/MD.0000000000037159

**Published:** 2024-03-08

**Authors:** Fen Li, Jie Zhao, Huanan Duan, Haixi Zhang, Lin Zhang, Liangyun Zhao, Yan Wen, Xuezhong Gu

**Affiliations:** a Department of Hematology, The First People’s Hospital of Yunnan Province, Yunnan Province Clinical Research Center for Hematologic Disease, Yunnan Province Clinical Center for Hematologic Disease, Kunming, 650032 Yunnan, China; b Department of Hematology, The Affiliated Hospital of Kunming University of Science and Technology, Kunming, 650032 Yunnan, China.

**Keywords:** chronic myelomonocytic leukemia, hematological malignancies, pyoderma gangrenosum

## Abstract

**Introduction:**

Pyoderma gangrenosum (PG) is a rare noninfectious neutrophilic skin disease. The diagnosis of PG is mainly based on clinical manifestations. Therefore, the clinical features of PG are important for confirming the diagnosis of this disease. Herein, the clinical data of 2 young males with PG complicated with hematological malignancies were reported, and the literature were reviewed.

**Case presentation:**

The first case was a 22-year-old male who was admitted due to a systemic rash, headache, and fever. Physical examination showed black scabs on the skins of the extremities, trunk, scalp, and face. Biopsy of the skin lesion showed epidermal edema, spongy formation, neutrophil infiltration, acute and chronic inflammatory cell infiltration in the dermis, showing purulent inflammation with epidermal erosion. The bone marrow biopsy showed obviously active proliferation of nucleated cells, granulocytes at various stages, abnormal morphological neutrophils, and occasionally observed young red blood cells. The diagnosis of PG and chronic myelomonocytic leukemia (CMML-0) was made. The second case was a 28-year-old male who presented a swollen, painful right calf following injury and then developed ulcers on skin and soft tissues. Bone marrow biopsy showed obviously active nucleated cell proliferation, suggesting a myeloid tumor. He was also diagnosed with PG and hematological malignancies. They both received hormone and antiinfection therapy. After treatment, their body temperature, infection, and skin lesions were improved. However, both of them were readmitted and had a poor prognosis.

**Conclusions:**

PG may be associated with hematological malignancies. For patients with typical skin lesions and obvious abnormal blood routines, it is necessary to investigate the possibility of PG with hematological malignancies.

## 1. Introduction

Pyoderma gangrenosum (PG) is a chronic and rare refractory skin disease characterized by inflammatory papules, pustules, and latent ulcers.^[1]^ In the clinic, patients often are admitted due to symptoms of PG, such as obvious pustules, ulcers, and pain, and are easily misdiagnosed as infectious diseases. Early debridement and incision drainage are performed according to the principles of infectious disease treatment. This may trigger the homomorphic reaction of PG in the acute phase, further aggravate the skin lesions, increase the difficulty of diagnosis, and bring great pain to the patients. PG is characterized by neutrophil infiltration in histology. The etiology and pathogenesis of PG are currently unclear, and there is no clear diagnosis and treatment guide. The skin lesion morphology, laboratory examination, and histopathology have no obvious specificity, which is an exclusive diagnosis and is easy to misdiagnose. Clinically, it can be divided into 4 types: ulcer, pustule, blister, and proliferative type. Ulcerative PG belongs to the most common of the 4 types, also known as classic PG, which was first reported by Brunsting et al in 1930. It can rapidly develop from a nodule or superficial hemorrhagic pustule to a painful ulcer, with central liquefaction necrosis, and a raised edge with a purple-red color. Irregular cribriform scars are often formed after the skin lesions are healed and are often accompanied by systemic diseases. In this report, we described the clinical data of 2 young males with PG complicated with hematological malignancies and reviewed the literature.

## 2. Case report

### 2.1. Case 1

**Figure 1.**
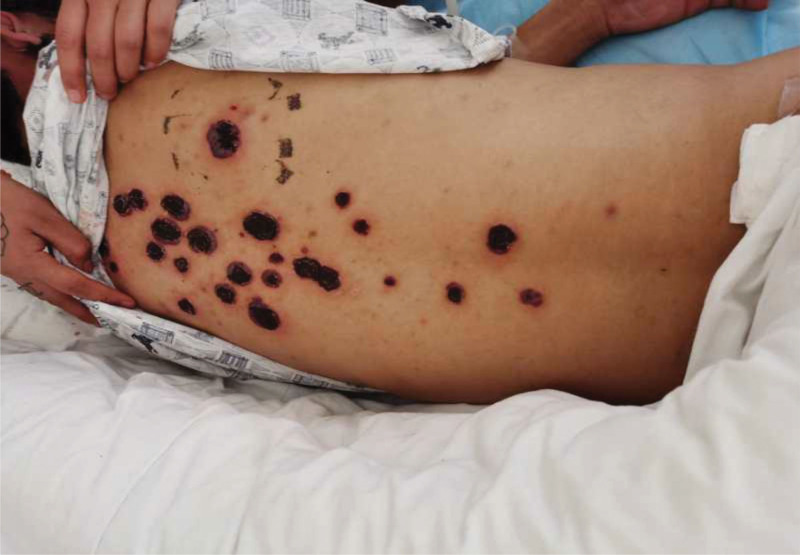
General appearance of skin lesion in case 1. Various size black scabs with slight flushing around were found on extremities, trunk, scalp, and face, with the largest of 4 × 4 cm.

A 22-year-old male was admitted due to a systemic rash for more than 10 days, headache for 1 week, and fever. Physical examination showed black scabs of various sizes with slight flushing around on extremities, trunk, scalp, and face, with the largest size of 4 × 4 cm (Fig. 1). The scattered ulcers were found on the oral mucosa and tongue tip. Blood test showed that white blood cell (WBC): 19.87*10^9^/L, BASO#: 0.05*10^9^/L, Hb: 106 g/L, PLT: 59*10^9^/L, RET#: 27.6*10^9^/L, PCT: 65.42 ng/mL, IL-6 > 8400 pg/mL, AST: 32 U/L, ALT: 61 U/L, creatinine: 132 µmol/L. The first bone marrow aspiration and biopsy showed obviously active proliferation of nucleated cells, granulocytes at various stages, abnormal morphological neutrophils, and occasionally observed young red blood cells. The proportion of monocytes accounted for 60% and blast cells accounted for 2.0%. The distribution of WBCs increased significantly, including granulocytes at various stages and with abnormal morphological. The proportion of monocytes accounted for 54%. There were no blasts. Mature red blood cells showed uneven morphological and size. The distribution of platelets decreased. A primary diagnosis of chronic myelomonocytic leukemia (CMML-0) was made. The second bone marrow puncture on 4 days later showed active bone marrow nucleated cell proliferation, G/E = 50.5:1, granulocyte hyperplasia, and granulocytes at various stages with abnormal morphology. Blasts accounted for 1.5%, and abnormal monocytes accounted for 32%. Biopsy showed silver stain (−), iron staining (+), MF grade 0, bone marrow 3-line hyperplasia, and an increased proportion of monocytes. Immunophenotyping: the monocyte population (R4 population) increased, accounting for 80.5%; R2 population accounted for about 6.4%, which expressed MPO, CD38, CD64, and CD15, some expressed HLA-DR, CD33, CD13, CD11b, and a small part expressed CD34; R1 population was considered as the granulocyte population, which showed that the SS was reduced and the antigen expression was disordered. Bone marrow chromosomes: 46,XY,del(3)(q12),?der(4)t(1;4)(q21;q32),del(13)(q21)[9]/46,XY[1]. Leukemia gene screening was negative. Myeloid gene mutations: RUNX1 44.14%, U2AF1 40.74%, PHF6 (c.663_668clclims13) 6.3%, PHF6 (c.725G>A) 73.26%; myelodysplastic syndrome (MDS)-FISH were all negative. Chest and abdomen CT scans showed lung infection and enlarged liver. Multiple blood cultures were (aerobic + anaerobic) negative. Biopsy of the skin lesion on the left back showed epidermal edema, spongy formation, neutrophil infiltration, and a large number of acute and chronic inflammatory cell infiltration in the dermis, showing purulent inflammation with epidermal erosion. He had repeated fever after admission, with the highest body temperature of 39.5°C. There was no improvement after 1 week of antiinfection. PG combined with CMML was considered. Therefore, prednisone 60 mg + thalidomide 150 mg was administrated on the basis of antiinfection. The temperature gradually stabilized. The pain of skin lesions was gradually relieved with scabs. Headaches were basically relieved. Platelet count was gradually increased and returned to normal level. Hemoglobin was slowly decreased to moderate anemia. WBCs were normal, and liver and kidney functions were improved. Later, he was transferred to the dermatology department of another tertiary hospital, and a biopsy of the skin lesion showed superficial necrosis and scab formation with an underlying ulcer and a large number of neutrophil infiltration, suggesting PG. He finally died of intestinal obstruction and sepsis.

## 3. Case 2

**Figure 2.**
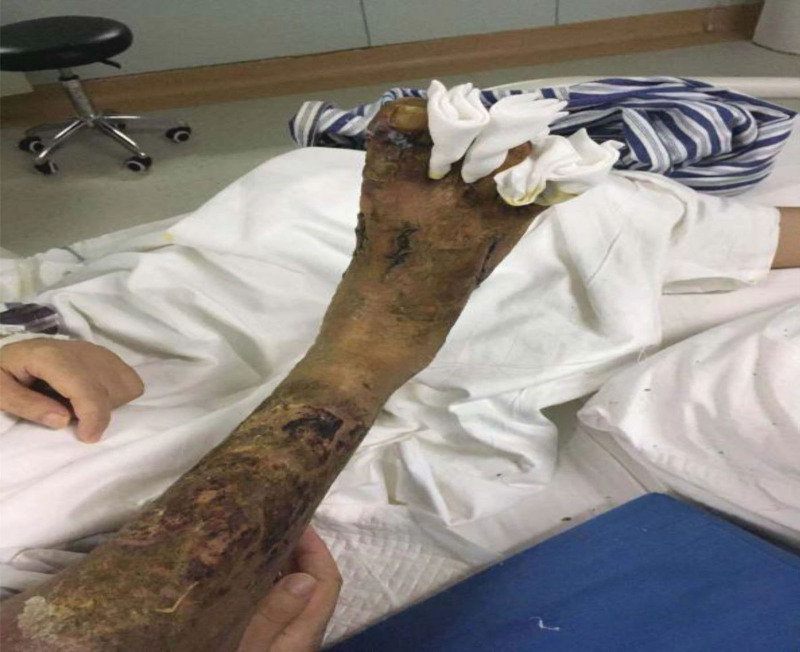
General appearance of wound in Case 2. A 10.0 × 8.0 cm superficial wound in the middle section of the anterior side of the calf, with partial skin and soft tissue defects, swelling of the surrounding soft tissue, high skin temperature, and a large amount of purulent secretions and necrotic tissue on the surface of the wound.

A 28-year-old male presented with a swollen, painful right calf following an injury. On day 12 postinjury, he had a fever with a max temperature of 40.5°C, increased swelling and pain of the right calf, and ulcers on skin and soft tissues. Bone marrow aspiration and biopsy at the local hospital showed the possibility of acute myeloid leukemia and MDS. Then he was transferred to the emergency department of our hospital. In the middle section of the anterior side of the calf, there was a 10.0 × 8.0 cm superficial wound with partial skin and soft tissue defects, swelling of the surrounding soft tissue, high skin temperature, and a large amount of purulent secretions and necrotic tissue on the surface of the wound (Fig. 2). WBC: 18.55 × 10^9^/L, N: 14.27 × 10^9^/L, HBG: 132 g/L, PLT: 221 × 10^9^/L. On the day of admission, a right calf debridement and negative pressure suction” was performed. After 2 weeks, the wound was improved, and the negative pressure drainage material was removed. The wound granulation tissue grew well; the extremity blood circulation was good, and the body temperature gradually returned to normal. Two days later, the right foot reappeared swelling. MRI showed diffuse edema of the skin, subcutaneous soft tissue of the right foot, and plantar muscle groups, unregular skin in some areas, infections in the subcutaneous tissue, muscle, and surrounding areas of the first and fifth toes, and the anterior part of the planta, and, a small amount of fluid in the right ankle joint cavity, suggesting compartment syndrome. Therefore, the right foot incision and decompression were performed immediately. After the operation, WBC count was continually increased, with the highest value of 97.64 × 10^9^/L. The body temperature was as high as 41°C, and there was no improvement after antiinfection treatment. The first bone marrow aspiration and biopsy showed obviously active nucleated cell proliferation. Blasts accounted for 16.5%, indicating the possibility of leukemia-like bone marrow and the possibility of hematopoietic system malignancy. The second bone marrow aspiration and biopsy showed extremely active nuclear cell proliferation, with blast cells accounting for 18.5%, suggesting myeloid tumors (possibly acute myeloid leukemia) complicating with infection. Immunophenotyping: R2 group accounted for about 12.2%. Cells in this group expressed CD45, CD13, CD33, HLA-DR; some expressed CD123, CD38, CD64, and MPO; a small part expressed CD117. The Granulocyte population showed a disorder of antigen expression and SS decrease, and R2 population was considered to be a myeloid proto-blast cell population, suggesting a myeloid tumor. The third bone marrow aspiration and biopsy showed obviously active proliferation of nucleated cells with 12.5% of blast cells. Bone marrow biopsy showed silver staining (−), iron staining (++), vigorous bone marrow granulosa hyperplasia, and focally distribution of blasts. Thus, myeloid tumors cannot be excluded. Myeloproliferative neoplasm (MPN) gene screening was negative. Leukemia gene screening: DEK-CAN positive. Bone marrow chromosome: 46, XY [10]. The results for blood culture (multiple times), wound secretions (multiple times), tissue culture, bone marrow culture during hospitalization, G test, and T-SPOT were all negative. Autoimmune-related antibodies were all negative. PG combined with myeloid tumor, pulmonary infection, and skin and soft tissue infection was considered. On the basis of antiinfection, intravenous infusion of methylprednisolone 40 mg, thalidomide 150 mg oral immunotherapy, hydroxyurea cell reduction, and other treatments were performed, and the body temperature was gradually returned to normal. The WBC was gradually returned to normal. The lung infection was controlled, and the skin lesions gradually improved. The patient was discharged after his condition was stable. Two weeks later, he was readmitted due to fever, with WBC: 2.8 × 10^9^/L, N: 0.95 × 10^9^/L, HBG: 82 g/L, PLT: 102 × 10^9^/L. A bone marrow biopsy was suggested, but the family finally gave up treatment.

## 4. Discussion

PG is a rare neutrophilic skin disease characterized by chronic and painful ulcers, with a variety of different clinical manifestations, and often accompanied by systemic diseases.^[1]^ The etiology of PG is not clear, which may be related to immune system disorders, neutrophil abnormalities, and environment and genetic factors.^[2,3]^ Since inflammatory infiltration of neutrophils mainly occurs in the lesion, PG is classified as a neutrophil lesion.^[4]^ The inflammatory factor IL-6, which plays an important role in activating and accumulating neutrophils, is significantly increased in the lesions of PG patients.^[5]^ In this study, IL-6 was significantly increased in case 1, which was greater than the upper limit of detection. However, the IL-6 data were lacking in case 2.

Common symptoms of PG include vascular occlusion or venous congestion ulcers, vasculitis, infection, and malignant tumors.^[6]^ There are many types of PG, including ulcerative, bullous PG, and postoperative PG, etc. Ulcerative PG is the most common classic subtype, mostly found in the lower limbs. Ulcer type and bullous type are more likely to be accompanied by blood system diseases (acute leukemia, myeloproliferative disease, MDS, lymphoma, etc).^[2,7]^ In this study, for case 1, PG was accompanied by CMML. Postoperative PG usually develops around 11 days after surgery and is usually misdiagnosed as wound infection.^[8]^ Case 2 of this study was a typical postoperative PG.

PG has no specific laboratory or histopathological markers, and its clinical manifestations are diverse. The clinical similarity with other skin diseases and various related systemic diseases makes the diagnosis of PG extremely challenging.^[9]^ Due to a lack of effective diagnostic criteria, PG is an exclusive diagnosis.^[10,11]^ In this study, case 1 had PG complicated with CMML. To the best of our knowledge, this is the first report of PG complicated with CMML. In 2016, the WHO classified CMML as MDS/MPN.^[12]^ Skin infiltration (cutaneous leukemia) can be the initial manifestation of CMML^[13,14]^ Patients with PG combined with CMML need to be differentiated from cutaneous leukemia. The blood system diagnosis of case 2 was unclear. The bone marrow biopsy 3 times showed blast cells accounted for 12.5%, 16.5%, and 18.5%, respectively, accompanied by DEK-CAN fusion gene. This DEK-CAN fusion gene is more common in acute myeloid leukemia and also in MDS patients who are usually young and have a poor prognosis.^[15]^ The immunophenotype of patients with DEK-CAN fusion gene often has the expression CD13, CD33, CD34, CD38, CD45, and HLA-DR.^[16,17]^ Case 2 in this study also had a similar phenotype expression. Currently, there is no literature reporting the detection of the DEK-CAN fusion gene in other blood system diseases or healthy people.

PG complicated with systemic diseases generally has a poor prognosis, and systemic diseases need to be treated at the same time. To date, there are probably dozens of cases of PG combined with leukemia reported.^[18,19]^ However, there is no clear evidence to explain the mutual pathogenic mode of the 2 diseases. Some consider that PG is secondary to leukemia,^[20]^ while others believe that the occurrence or recurrence of leukemia is related to the recurrence of PG ulcers, and controlling leukemia can improve PG skin lesions.^[12,18]^ The focus of PG treatment is to reduce systemic inflammation. So far, there is no gold standard therapy for PG. Therefore, most treatments are planned according to expert opinions, case reports, and small cohort studies, which mainly depend on location, number and size of the lesion, extracutaneous involvement, potential systemic disease, and cost. Treatment of PG includes local treatment and systemic treatment, and local treatment includes corticosteroids and tacrolimus.^[21,22]^ The most common first-line drug for systemic therapy of PG is steroids or glucocorticoids.^[23,24]^ The combined use of glucocorticoids and immunosuppressive agents is mostly recommended.^[25]^ Two studies have confirmed that the use of hormones in combination with another immunomodulator in systemic therapy can achieve better results.^[26,27]^ Pain control is also an important treatment principle for PG and nonsteroidal anti-inflammatory drugs and opioids are recommended for pain control. Unfortunately, neither of these 2 patients in this study received hematological disease-related treatments. For patients with PG combined with hematological malignancies, it is not clear whether PG or hematological tumors should be treated first. In case 1, the treatment of PG was first administrated, and the rash improved, but the treatment opportunity was lost due to secondary infections. Case 2 also primarily received the treatments of PG, with a preferred efficacy, but finally gave up further treatment of hematological tumors. Therefore, the timing, method and focus of treatment for patients with PG complicated with hematological tumors are still issues that need to be further explored.

There are some limitations in the study. The number of in-cohort cases was not large enough to draw a conclusion about PG diagnosis and treatment protocols. Also, the correlation of PG with other co-morbidities needs further analysis.

In conclusion, PG is a rare neutrophilic skin disease with complicated etiology and unclear pathogenesis. Usually, patients with systemic diseases, skin lesions, postoperative wounds, and ulcers should be cautious of PG. Patients with PG should be cautious with systemic diseases and should receive treatments timely.

## Author contributions

Conceptualization: Fen Li, Jie Zhao, Xuezhong Gu.

Data curation: Fen Li.

Funding acquisition: Fen Li, Jie Zhao, Haixi Zhang, Liangyun Zhao, Yan Wen, Xuezhong Gu.

Investigation: Fen Li, Jie Zhao, Huanan Duan, Haixi Zhang, Lin Zhang, Liangyun Zhao, Yan Wen.

Validation: Fen Li.

Writing—original draft: Fen Li.

Writing—review & editing: Jie Zhao, Xuezhong Gu.

Resources: Huanan Duan, Haixi Zhang, Lin Zhang, Liangyun Zhao, Yan Wen.

Project administration: Xuezhong Gu.

Supervision: Xuezhong Gu.
